# Targeted Therapeutic Approaches for the Treatment of Cancer: The Future Is Bright

**DOI:** 10.3390/jpm15040141

**Published:** 2025-04-02

**Authors:** Matthew J. Hadfield, Benedito A. Carneiro, Liang Cheng

**Affiliations:** 1 Legorreta Cancer Center, Brown University, Providence, RI 02912, USA; benedito_carneiro@brown.edu; 2 Department of Pathology and Laboratory Medicine, Department of Surgery (Urology), Brown University Warren Alpert Medical School, the Legorreta Cancer Center at Brown University, and Brown University Health, Providence, RI 02912, USA

The last two decades have ushered in unprecedented advancements in the treatment of cancer. The development of drug targets based on the molecular makeup of tumors has resulted in multiple new classes of anti-cancer therapies and novel drug designs and mechanisms of action. The shift towards personalized therapy was predicated on the decoding of the cancer genome. The better delineation of driver genomic alterations and signaling networks has contributed to the unraveling of a multitude of new drug targets. This has been possible due to dramatic improvements in multiple fields including biotechnology, molecular genetics, and molecular sequencing. The integration of digital pathology, advanced tools for the characterization of tumor microenvironments, and target expression aligned with artificial intelligence are reshaping the future of precision medicine and drug development in oncology.

The molecular characterization of tumors has made possible the development of entirely new classes of anti-cancer therapies and these advancements have ushered in the era of targeted therapeutics, drugs that target specific genomic alterations and their downstream proteins. The successful therapeutic targeting of the Kristen rat sarcoma virus (KRAS) gene represents a relatively recent major achievement. For decades, KRAS was considered ‘undruggable’ due to the small size of the protein and its biochemically unfavorable binding sites [1]. This paradigm was reversed with the Food and Drug Administration (FDA)’s approval of the first KRAS inhibitor, sotorasib, for the treatment non-small-cell lung cancer (NSCLC) harboring KRAS G12C mutations in 2021, followed by the approval of adagrasib in 2022. Currently, several pan-KRAS inhibitors are in clinical trial development with promising preliminary results. To date, there are over 50 small molecule drugs targeting protein alterations across all solid tumors [2].

A better understanding of tumor biology changed the classification of malignancies and refined molecular subtypes based on novel targets. Specific histology-based definitions of malignancy have incorporated the molecular profile to better characterize tumors. There have now been several regulatory approvals for the tissue-agnostic utilization of drugs based on specific genomic alterations. These include larotrectinib and entrectinib for neurotrophic tyrosine receptor kinase (NTRK) fusions (2018), dabrafenib and trametinib for B-Raf-Protoco-Oncogene (BRAF) V600E mutated solid tumors (2022), and selpercatanib for rearranged during transfection (RET) fusion cancers (2022) [3].

The field of targeted therapies has contributed to the enhancement of the clinical impact of antibody–drug conjugates (ADCs) as a new class of drugs. Following a long development trajectory, the first ADCs were approved by the FDA in 2000. Increased bioengineering capabilities and molecular genetics have enabled the exploration of new targets and more efficient drug design, and this has led to over 25 FDA approvals at the present time, across multiple tumor types [4]. The best example of ADCs dramatically changing the landscape of standard-of-care treatment options is breast cancer, where trastuzumab–deruxtecan (T-DXd) redefined HER-2 positivity by demonstrating efficacy in the HER-2 subtype of tumors [5]. Following the trend of small-molecule inhibitors, T-Dxd became the first ADC to receive a tissue-agnostic approval in 2024 for tumors expressing HER-2 positivity.

Advancements in drug discovery have been augmented by profound improvements in biomarker testing capabilities. The utilization of computational pathology, now augmented by artificial intelligence, has enabled the better characterization of cell surface markers [6]. Additionally, large language models have made it possible to analyze vast amounts of unstructured patient data to better elucidate predictors of response and toxicity [7]. This unprecedented progress has called into question traditional study design and has led to innovative platforms for better, more efficient clinical trials. Perhaps the best example of this is the Project Optimus initiative by the US Food and Drug Administration, which aims to better identify efficacious dosing for targeted therapies and avoid unnecessary toxicity by escalating to higher doses without additional benefit [8].

Despite an ever-increasing number of therapeutic options for patients, significant hurdles still limit their broader adoption. Next-generation sequencing (NGS) remains underutilized despite being the standard of care for a multitude of tumors [9]. Multiple cancers lack effective targeted therapeutic options. The discovery of new biomarkers and the development of novel targeted therapies will heavily depend on shared resources from international consortiums as well as robust collaborations between industry and academic researchers.
